# GLI3: a mediator of genetic diseases, development and cancer

**DOI:** 10.1186/s12964-020-00540-x

**Published:** 2020-04-03

**Authors:** Stephan J. Matissek, Sherine F. Elsawa

**Affiliations:** Department of Molecular, Cellular and Biomedical Sciences, University of New Hampshire, 46 College Rd Rudman 291, Durham, NH 03824 USA

**Keywords:** GLI, GLI3, Hedgehog signaling, Development, Cancer, Genetic disease

## Abstract

The transcription factor GLI3 is a member of the Hedgehog (Hh/HH) signaling pathway that can exist as a full length (Gli3-FL/GLI3-FL) or repressor (Gli3-R/GLI3-R) form. In response to HH activation, GLI3-FL regulates HH genes by targeting the GLI1 promoter. In the absence of HH signaling, GLI3 is phosphorylated leading to its partial degradation and the generation of GLI3-R which represses HH functions. GLI3 is also involved in tissue development, immune cell development and cancer. The absence of *Gli3* in mice impaired brain and lung development and *GLI3* mutations in humans are the cause of Greig cephalopolysyndactyly (GCPS) and Pallister Hall syndromes (PHS). In the immune system GLI3 regulates B, T and NK-cells and may be involved in LPS-TLR4 signaling. In addition, GLI3 was found to be upregulated in multiple cancers and was found to positively regulate cancerous behavior such as anchorage-independent growth, angiogenesis, proliferation and migration with the exception in acute myeloid leukemia (AML) and medulloblastoma where GLI plays an anti-cancerous role. Finally, GLI3 is a target of microRNA. Here, we will review the biological significance of GLI3 and discuss gaps in our understanding of this molecule.

**Video Abstract**

**Video Abstract**

## Background

The *GLI* gene was first identified in humans as a highly expressed gene in human glioma [1]. Using cDNA probes for the zinc finger region of the *GLI* gene, Ruppert et al (1988), identified two additional GLI family members, *GLI2* and *GLI3* [2]. Further characterization of human GLI3 revealed it to be a 190 kDA protein located on chromosome 7p13 and binds to consensus sequences similar to those of GLI1 [3]. The most updated data on the National Center for Biotechnology Information (NCBI) and new publications, mapped human GLI3 to chromosome 7p14.1 (Gene ID:2737, 4]. *GLI3* was identified as a gene in which mutations in *GLI3* cause GCPS, a disease leading to craniofacial and limb maldevelopment. In a study by Vortkamp et al (1991), 2 translocations in *GLI3* were identified, which interrupt GLI3 expression and cause GCPS [5]. Point mutations in the human *GLI3* locus in GCPS patients were identified as a main cause of GCPS disease manifestation [6]. In 1996, GLI3 was described as a protein that is regulated in response to the sonic hedgehog (SHH) signaling pathway where it was described to compete in binding with GLI1 [7]. In the same study, GLI3 was characterized as a negative regulator of SHH signaling [7]. In the following year, GLI3 was recognized as the cause of PHS, a disease characterized by developmental malformations including polydactyly (extra digits) [8]. Follow-up studies described Gli3 as both an activator and repressor, similar to the Gli2 family member, in response to Shh signaling [9]. Since then, research on mouse and human Gli3/GLI3 mostly focused on its role in brain and limb development with certain exceptions of Gli3/GLI3’s role in angiogenesis, colorectal and liver cancer, TRAIL-dependent apoptosis and its role in regulating the IL-6/JAK2 pathway [10–14].

## Regulation and structure

### Hedgehog ligands and their function

The Hh signaling pathway plays a role in embryonic development and homeostasis of stem cells in normal tissues [15]. Dysregulations of Hh signaling cause genetic defects such as holoprosencephaly and polydactyly and are tightly linked to cancer development and progression [16, 17]. In addition, a role for Hh signaling in hematopoiesis and in the immune system has been described [18–20]. The Hh signaling pathway is activated by 3 ligands: Sonic Hedgehog (Shh/SHH), Indian Hedgehog (Ihh/IHH) or Desert Hedgehog (Dhh/DHH) (mouse/human respectively) [21]. These ligands are roughly 45 kDA with a N-terminal biologically active domain and an autocatalytic C-terminus, which is cleaved to generate the final Hh ligand form [22, 23]. After cleavage, a cholesterol moiety is added to the C-terminus and palmitate is linked to the N-terminus [24]. This allows exogenous Hh ligands to travel far distances to activate Hh signaling in various cells/tissues in the body [25]. Shh is the ligand with the highest expression and therefore is the major inducer of most Hh-related functions such as brain, limb and spinal cord development [26, 27]. Ihh was linked to chondrogenesis and negatively regulates chondrocyte differentiation [28]. Dhh null male mice are infertile while there was no visible effect in female mice suggesting a role for Dhh in spermatogenesis [29]. Additionally, Dhh was shown to play a role in peripheral nerve ensheathment [30]. Shh is mostly expressed in epithelia while Dhh is expressed in Schwann and Sertolli precursors and Ihh is expressed in the cartilage and in the gut [31]. All Hh ligands bind to the same receptor Ptch1 and initiate Hh-related signaling. However, Shh has been shown to be the most potent inducer of this pathway [32].

### Classic (canonical) Hh signaling

Many components known to be involved in vertebrate Hh signaling were initially identified in *Drosophila*. The main components of Hh signaling in *Drosophila* are 1) Patched (Ptc) a 12-transmembrane protein which binds Hh ligand; 2) Smoothened (Smo), a receptor that is repressed by Ptc and released to activate the pathway once Hh ligand binds Ptc; and 3) Cubitus interruptus (Ci) which is the *Drosophila* analog of Gli proteins in vertebrates [33]. In the absence of Hh signaling Ci, Costal-2 (cos-2) and Fused (Fu) form the Hedgehog Signaling complex (HSC). This leads to proteasomal degradation of Ci from a 155 to 75 kDA form through phosphorylation of Protein Kinase A (Pka/PKA), Glycogen synthase kinase-3 beta (Gsk3β/GSK3β) and Casein Kinase 1 (Ck-1/CK-1) [34]. The degraded form of Ci subsequently acts as a repressor of Hh signaling. Once Hh signaling is activated by ligand binding to Ptc, and inhibits its repressor function, Smo is activated which leads to stabilization of Ci and expression of Hh related genes. The signaling cascade is well conserved between invertebrates and vertebrates, therefore the information gained from *Drosophila* is useful in predicting the signaling events in mice and humans.

#### In mammals

In mammals, Hh signaling occurs at the primary cilia of cells [35]. The intraflagellar transport (IFT) proteins: Intraflagellar transport protein 172 homolog (Ift172), Kinesin-like protein KIF3A (Kif3a), dynein cytoplasmic heavy chain 2 (Dnchc2) and Polaris, are extremely important for the formation and function of cilia, and are essential for the regulation of Hh activation or inactivation [36–38]. In addition, the kinesin-like protein Kif7, which is involved in cilia tip assembly, was shown to be essential for proper activation of this pathway and is therefore involved in positively and negatively regulating Hh-dependent functions [39, 40]. Several co-receptors for Ptch1 have been identified and were shown to be indispensable for proper Hh function. These include growth-arrest-specific 1 (Gas1), CAM-related/downregulated by oncogenes (Cdo), and brother of Cdo (Boc) which positively regulate Hh-related signaling events [41]. In addition, the Hh interaction protein (Hhip) binds and sequesters all three Hh ligands and therefore acts as a negative regulator of the Hh pathway [42].

In the absence of binding of one of the Hh ligands (Shh, Dhh or Ihh) to Ptch1, Smo is localized intracellularly [43]. The mechanism by which Ptch1 inhibits Smo is not completely understood. It was hypothesized to occur by either binding of a Smo activator or delivery of a Smo small molecule inhibitor [44]. Another hypothesis suggested that in the absence of Hh ligand, Ptch1 translocates to the base of the primary cilia and inhibits localization of Smo to the cilia [45, 46]. However, further experiments are needed to elucidate the mechanism by which Ptch1 inhibits Smo. Without SMO being activated, suppressor of fused (Sufu/SUFU) sequesters GLI transcription factors in the cytoplasm [47]. These GLI proteins are essential effectors of the HH pathway. There are 3 members of the GLI family of transcription factors: GLI1, GLI2 and GLI3. In the absence of Hh activation by ligand binding to Ptch1, Sufu is phosphorylated and recruits G-protein coupled receptor 161 (Gpr161/GPR161), PKA, GSK3β, and beta-tranducin repeat containing E3 ubiquitin protein ligase (βTrCP) complex to proteolytically cleave Gli2 and Gli3 into smaller proteins with a repressor function [48, 49]. Ck-1 negatively regulates Hh signaling in *Drosophila* [50]. Since many Hh signaling molecules in *Drosophila* are evolutionary conserved in mammals, it is probable that Ck-1 negatively regulates mammalian HH signaling as well. In fact, mutations of conserved Ck-1 sequences from *Drosophila* showed less phosphorylation of mammalian GLI3 and less GLI3 processing into its repressor form [51]. However, based on our current understanding, it remain unclear what role CK-1 plays in vertebrate HH signaling since there are also reports of positive regulation of HH signaling by CK-1α and CK-1ε [52–54]. Interestingly, in *Drosophila*, inhibition of Ck-1α or Ck-1ε alone did not significantly affect Ci processing [55]. However, when both Ck-1 subtypes were inhibited, a significant decrease of Ci repressor was observed. Therefore, it is probable that CK-1α and CK-1ε alone positively regulate HH signaling, while when forming an alpha-epsilon complex, they negatively regulate this pathway by phosphorylating GLI3. This however, is highly speculative, and more research is needed to validate the role of CK-1 in HH signaling. GLI1 is not cleaved in this process but completely degraded [56]. While Gli2 is not very stable in its repressor form and completely degrades after cleavage, the Gli3 repressor form is more stable and translocates to the nucleus where it inhibits Hh-related responses [57]. As such, in the context of Hh/HH signaling, GLI3 is known to be the most potent inhibitor of HH signaling within the GLI family.

**Fig. 1:**
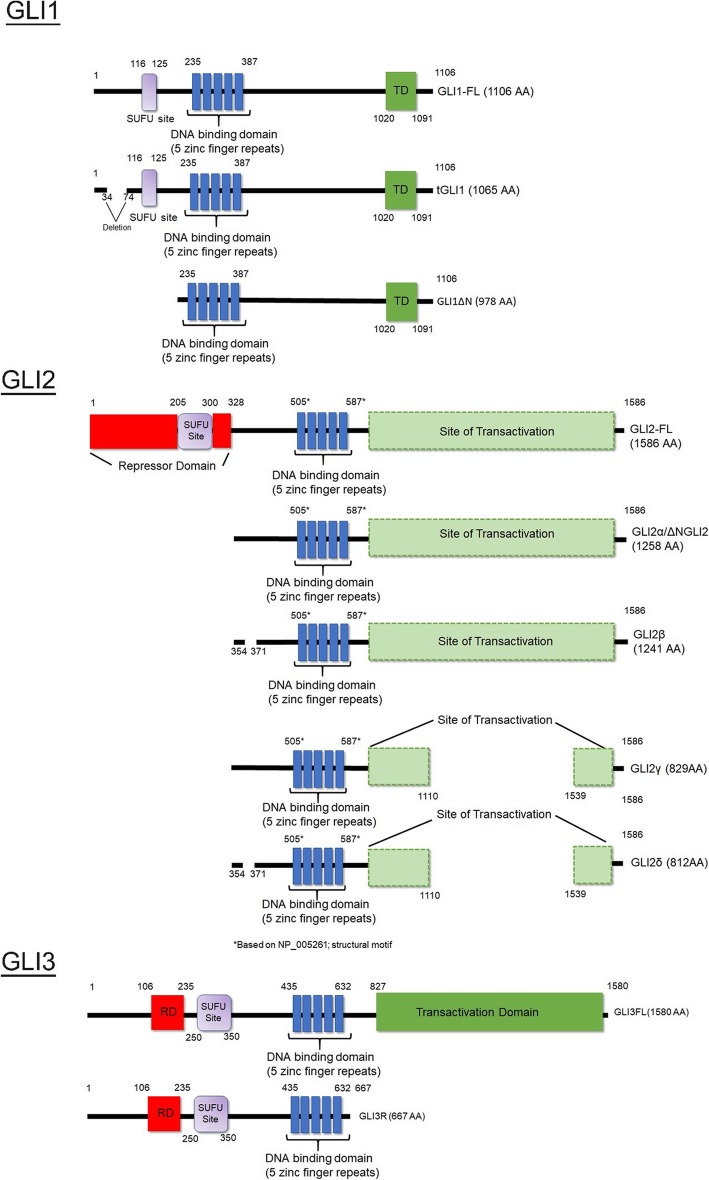
Biochemical domains of GLI family members. Schematic diagram of the biochemical domains of human GLI1, GLI2 and GLI3 showing the relative repressor (RD), DNA binding and transactivation (TD) domains. HH-related transcriptional activator GLI1 is the smallest protein of the GLI family with 1106 AA (GLI2: 1586 AA; GLI3: 1580 AA). In comparison to GLI2 and GLI3, GLI1 lacks a transcriptional repressor domain. GLI1 and GLI2 isoforms have been described. There is no direct evidence to describe the exact position of the transactivation domain and zinc finger region of human GLI2

Upon ligand binding to Ptch1, Smo translocates to the primary cilia, a process mediated by β-arrestin [58]. This leads to the association of Smo with Ellis-van Creveld syndrome protein/Ellis-van Creveld syndrome protein 2 (Evc/Evc2) complex (a complex that regulates chondrocyte proliferation and differentiation of osteoblasts) in which Sufu-Gli2/3 complex moves to the tip of the cilia where Sufu dissociates from Gli2/3 [57, 59, 60]. Sufu is then degraded and Gli2/3 full-length accumulate at the ciliary tip and can translocate into the nucleus to activate a Hh-related gene expression program [49, 61]. Gli1, Gli2-FL, and Gli3-FL have been shown to induce Hh related gene functions. Because GLI2-FL and GLI3-FL induce GLI1 expression, GLI1 therefore acts as an amplifier of the HH signal [62]. However, Gli2 is considered the most potent activator of Hh signaling [9, 63]. All members of the GLI protein family (GLI1, GLI2 and GLI3) contain five zinc finger domains which are very similar to those of Ci [64]. Although all GLI members share biochemical domains, GLI1 does not contain a N-terminal repressor domain which is present in both GLI2 and GLI3 (Fig. 1). Therefore, biochemically, GLI1 can only act as transcriptional activator while GLI2 and GLI3 can potentially act as transcriptional activators or repressors.

In humans, different isoforms of GLI1 and GLI2 have been reported (Fig. 1). To date, there are no reports of different GLI3 isoforms in humans. GLI1 has three isoforms: isoform 1 is 1106 amino acids (AA) long and is considered the full length GLI1 protein (NP_005260.1). GLI1 isoform 2 sequence lacks exons 2 and 3, which leads to a truncated GLI1ΔN form (978 AA) (NP_001153517.1). This isoform was reported in healthy and malignant tissue and showed no binding to SUFU [65]. The third isoform tGLI1 is a cancer-associated form of GLI1 [66], and is not detected in normal cells but is found in glioblastoma multiforme (GBM) and other cancers. It lacks exon 3 and part of exon 4 (1065 AA), but retains most of its function; however, it is associated with increased motility and invasiveness [66]. To date, four GLI2 isoforms have been described, in addition to the full length GLI2 (1586 AA) [67, 68]. These isoforms include GLI2α (1258 AA), GLI2β (1241 AA), GLI2γ (829 AA) and GLI2δ (812 AA). GLI2α is also known as ΔNGLI2 and shows significantly higher activity *in vitro* than GLI2 full length [69]. GLI2β mRNA was identified to be highly expressed in basal cell carcinoma and both GLI2α and GLI2β were shown to regulate the expression of the human T cell lymphoma virus 1 gene [67, 70]. To date, no functions for GLI2γ and GLI2δ have been reported.

### Non-classical (non-canonical) HH signaling

PTCH1 and SMO are major regulators of the canonical HH signaling pathway and therefore of GLI transcription factors. However, several studies report that GLIs (especially GLI1 and GLI2) can be regulated by the cross talk with other signaling pathways in various types of cancers including melanoma, gastric cancer, colon cancer, multiple myeloma, medulloblastoma, pancreatic cancer, glioblastoma, and osteosarcoma [71–78].

The EGFR-RAS-RAF-MEK1 pathway has been frequently investigated in regulating GLI1 and GLI2 expression levels. Constitutively active MEK1 and oncogenic KRAS^V12^ induce GLI1 protein expression and activity [71, 79]. In addition to upregulating positive regulators of HH signaling, GLI2 was shown to be stabilized upon activation of EGF-ERK1/2 signaling [80]. Utilization of the A3 adenosine receptor agonist, resulted in a decrease in breast cancer stem cell survival which correlated with a decrease in activated ERK1/2 and GLI1 [81, 82]. Therefore, EGFR signaling through MAPK can regulate GLI1 and GLI2 at the level of expression and activity.

The PI3K pathway has been closely linked to HH-related functions. Several studies suggest a role of PI3K-AKT axis in the regulation of GLI1/2 transcriptional activators [83–85]. PI3K-AKT activity induced an increase in the expression of GLI1 and GLI2 in renal cell carcinoma and chronic lymphocytic leukemia (CLL), leading to increased cancer cell growth and progression [83, 84]. The regulation of GLI by PI3K-AKT axis was shown to be induced by CCL5-CCR3 signaling in the tumor microenvironment (TME) as well [85]. In addition, the interaction between PI3K and HH has been reported to be important for tamoxifen resistant breast cancer and combined targeting of both GLI1/2 (using the GLI antagonist GANT61) and PI3K/mTOR (using PI103) synergistically increased apoptosis and reduced tumor growth [86, 87].

In addition to ERK1/2 and PI3K, TGF-β has been shown to be involved in regulating GLI1/2 expression without the involvement of SMO signaling [88–92]. In the last few years several studies have reported a role for the TGF-β-GLI1/2 axis, partially Smad3 dependent, in fibroblast activation, melanoma, stroke (ischemia/reperfusion injury), hepatocellular carcinoma, and gastric cancer [88–92]. In these studies, both GLI1 and GLI2 regulate tumor growth, survival and epithelial-to-mesenchymal transition (EMT) upon stimulation by TGF-β1/2 in a SMO independent manner. Interestingly, TGF-β2, an inducer of nephrons (a unit required for functional kidneys) lies downstream of GLI3-R [93]. This suggests that a negative feedback loop exists between TGF-β-dependent HH activation in which GLI1 and GLI2 act as positive regulators while GLI3 acts as a negative regulator. However, more experimental evidence is needed to validate this hypothesis. Additionally, in a pan cancer analysis, a positive correlation between TGF-β-related gene expression and GLI1 and GLI2 expression was observed [94]. Notably, GLI1/2 expression was more of a prognostic factor for TGF-β and EMT-related genes than for HH-related genes, which might suggest that GLI1/2 regulate tumor formation by regulating TGF-β-related gene expression rather than HH-induced gene expression.

Protein kinase C (PKC), a known stimulator of cell proliferation, was also reported to regulate GLI expression and function [95, 96]. Although PKC was shown to phosphorylate and activate SMO at the beginning of the HH signaling cascade, it was also reported to be able to compensate for SMO inhibition and induce GLI activation [97, 98]. Interestingly, PKC-mediated activation of GLI proteins occurs independently of primary cilia and MEK-1 in NIH3T3 cells [99]. PKC also positively regulates GLI1-HDAC1 association [100] and targeting HDAC1 with the HDAC inhibitor Vorinostat, only resulted in a decrease in cell proliferation at high inhibitor doses. In combination with Vorionstat, PKC inhibition enhanced the therapeutic effect and increased the therapeutic window in basal cell carcinoma [101].

The intracellular membrane trafficking protein Rab23, the serine/threonine kinase involved in cell proliferation and survival CK2α, the inhibitor of transcription SLTM, the proliferative proteins Dual Specificity Tyrosine Phosphorylation Regulation Kinases 1 (DYRK1) and DYRK2, the metabolic proteins MAP 3K10 and AMPK all regulate GLI1 expression and function as well [102–106]. These regulators are, in some way, involved in cancer growth and progression, which shows the importance of studying the crosstalk between HH signaling and other pathways, particularly in GLI-directed therapeutic approaches.

Most studies investigating non-canonical HH signaling primarily focus on GLI1 and GLI2 and oftentimes did not consider GLI3 in their experimental approach. This is understandable since GLI1 and GLI2 are positive regulators of HH ligand-induced gene expression and can therefore be considered major drivers of HH-related genes [107]. However, since various studies describe GLI activation by other signaling pathways which are activated by different ligands (other than SHH, IHH or DHH), the regulation of GLIs might be different in comparison to their regulation in the canonical HH pathway. For example, PKC has been shown to activate GLIs in a cilia-independent manner and additionally, pan cancer analysis revealed that GLIs account for TGF-β-induced gene expression rather than genes induced by classical HH pathway [94, 99]. In addition, there are multiple examples that are discussed in this review of the role of GLI3 in development, in the immune system and in cancer where GLI3 often acts as a positive regulator of those pathways, although GLI3 did not show potent HH-gene induction in the canonical HH pathway. It might be that activation, regulation and function of GLIs varies depending on which pathway triggers them, which raises the question of whether or not we can apply the principles of knowledge of the canonical pathway to the non-canonical pathways. Could it be that GLI3-FL acts as a more potent gene activator than GLI2-FL depending on which non-canonical pathway is active? Are post translational changes involved in increasing GLI3-FL function or are GLI3 mRNA levels induced? Is GLI3-FL processing into its repressor form always dependent on PKA, GSK3β or βTrCP or could these factors be substituted, differentially regulated or inhibited in different pathways of non-canonical HH signaling? This report aims to give insights into different cellular functions of GLI3 which are oftentimes in crosstalk with other important signaling pathways.

### Regulation of GLI3

#### Gene locus and domains of mouse and human Gli3/GLI3

**Fig. 2:**
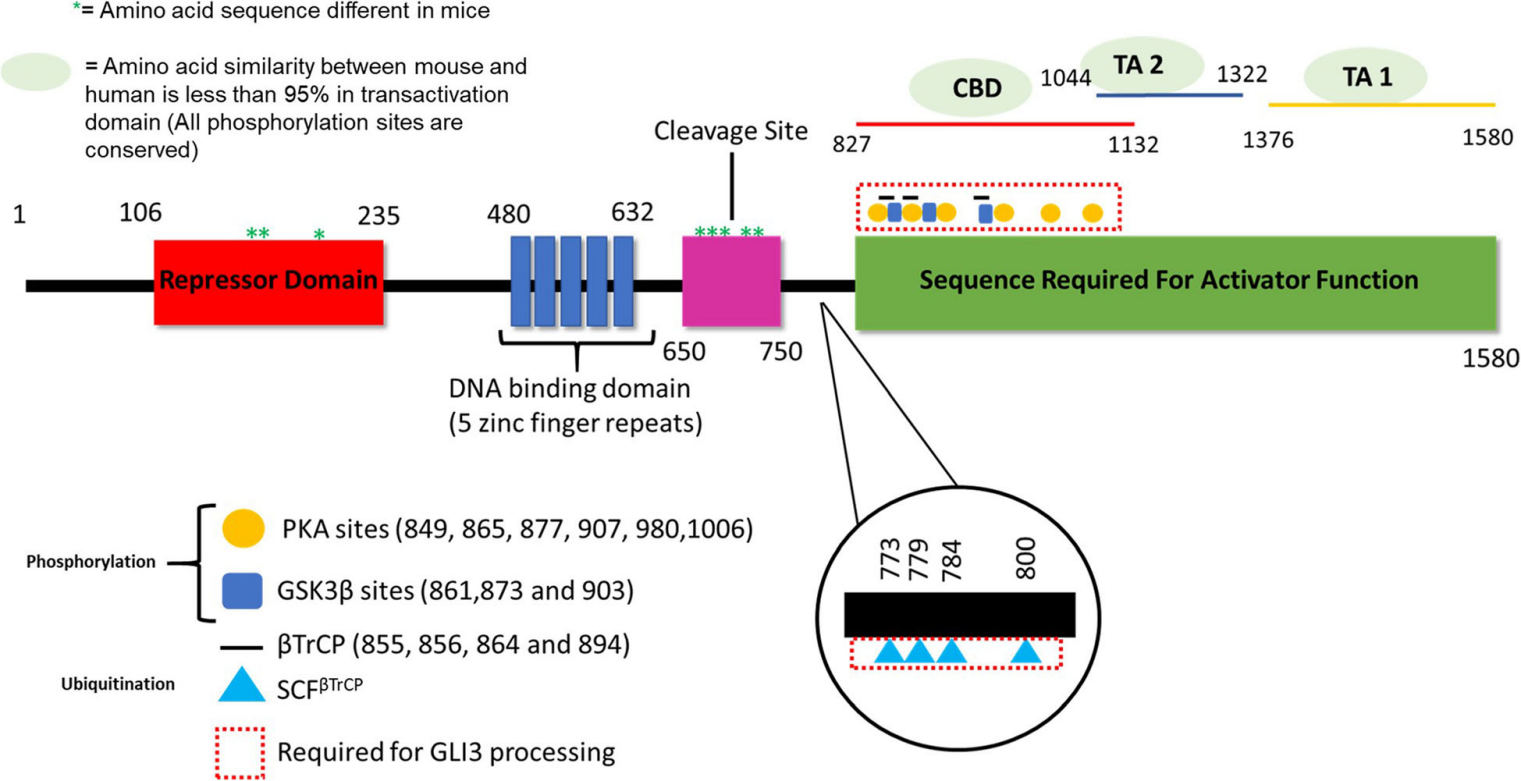
Biochemical domains of human GLI3 with posttranslational modifications. GLI3 can be phosphorylated by PKA and GSK3β which leads to binding by βTrCP and ubiquitination by SCF^TrCP^. Indicated phosphorylation and ubiquitination sites are shown and are crucial for GLI3 processing into its repressor form (red box). βTrCP binds GLI3 at the C- and N-terminus and at its core domain (activation domain; green). Only binding to GLI3 core domain is important for GLI3 processing into GLI3-R. Processing site to generate Gli3-R and zinc finger domain are completely conserved between mouse and human. Differences in amino acid sequences between mice and human are represented by asterisk and oval structure

Several publications have reported that human GLI3 is located on chromosome 7p13 [5, 8, 23]. However as mentioned earlier, recent updates on NCBI have mapped human GLI3 to chromosome 7p14.1 (Gene ID: 2737) [4, 108, 109]. The human *GLI3* gene is 276261 bp in length (NC_000007.14) and the mRNA sequence is composed of 15 exons that are 8405 bp in length (16 Dec, 2019) (NM_000168). However, the consensus coding sequence (CCDS) is 4743 nucleotides (nt) (nt 282-5024) with a transcription start site located in exon 2 (CCDS5465.1). Due to an error in sequencing, older literature characterized GLI3-FL to be 1596 AA in size which was later corrected to 1580 AA [110]. This is also listed in the protein database entry NP_000159.3 which shows GLI3 protein to be 1580 AA with a molecular weight of 190 kDa (GLI3-FL)(NP_000159.3). Human GLI3 contains a N-terminal repressor domain which is mapped to AA 106-235 (exons 3-6, mRNA nt 649-986) [111]. GLI3 also has a 5 zinc finger DNA-binding domain (AA 480-632 that span exons 10-13; nt 1719-2177), and a C-terminal transcriptional activation domain that is dependent on CREB-binding protein (CBP) binding (AA 827-1132 in exon 15; nt 2760-3677) (Fig. 2) in addition to transactivation domain 2 (TA2)(AA 1044-1322 in exon 15; mRNA nt 3411-4247) and transactivation domain 1 (TA1)(AA 1376-1580 in exon 15; mRNA nt 4407-5024) [62, 112]. The proteasomal cleavage site spans exons 13 and 14 (AA 650-750; nt 2229-2531) which leads to the truncated GLI3-R (677 AA with a molecular weight of 83 kDa) [113–115]. In addition to other references listed, mapping of DNA binding domain, cleavage site and activator domain was performed with the help of the publication by Krauß et al (2009) [116]. Although it can be extrapolated that the stated AA range corresponding to each domain is accurate, studies other than that describing the repressor domain [111], that precisely define GLI3’s biochemical domains, are lacking.

**Fig. 3:**
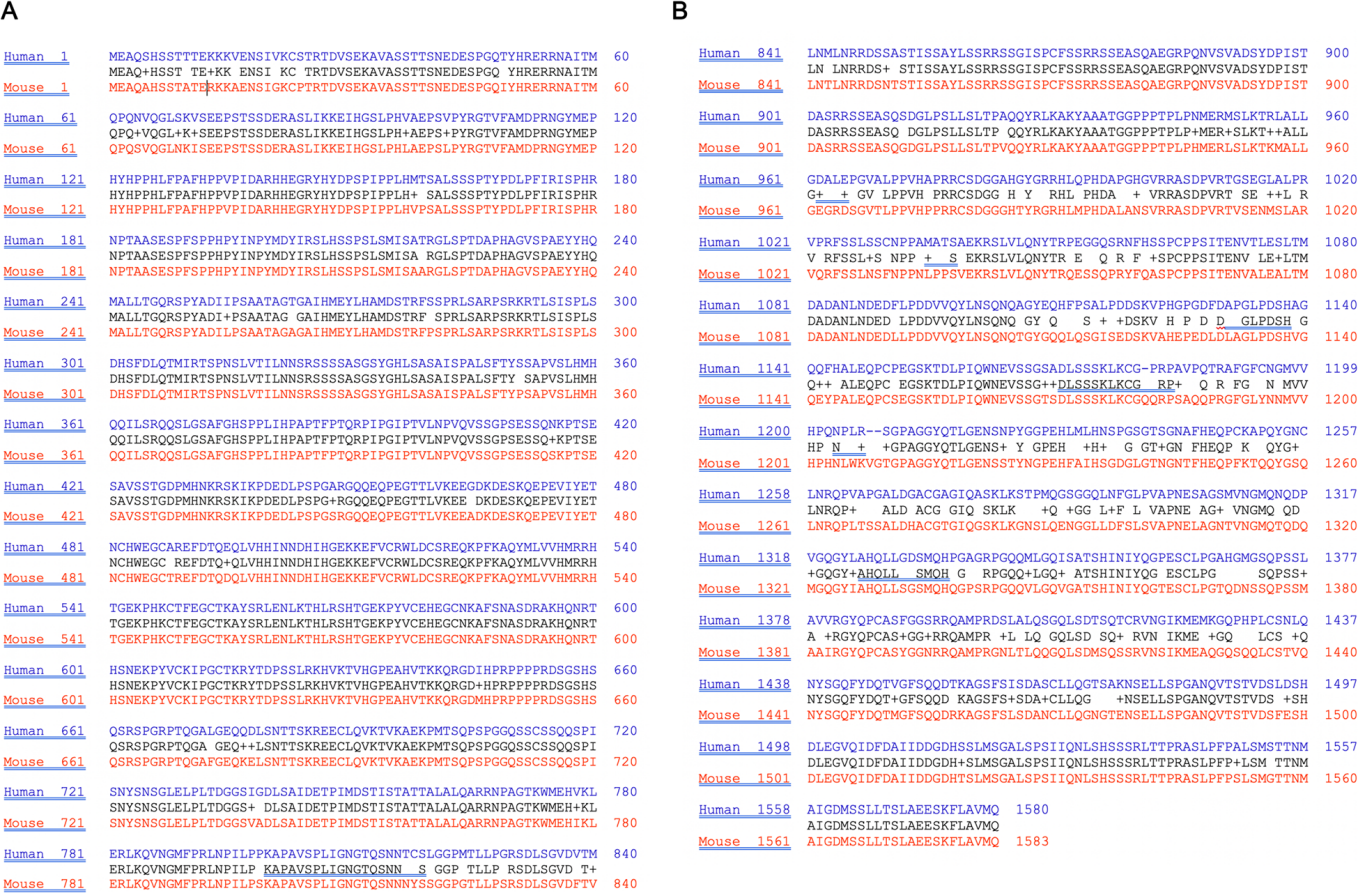
Alignment of human and mouse GLI3/Gli3 protein sequences. Alignment of human (NP_000159.3) and mouse (NP_032156.2) GLI3/Gli3 shows 86 % similarity in amino acid sequence

In mice, *Gli3* is located on chromosome 13 with 266304 bp in length (NC_000079) (last update 19-OCT-2010). The mRNA sequence is 8427 nt long and comprises 15 exons (NM_008130) (last update: 30-Dec-2019). Between exons 2 and 15 (nt 431-5182) lies the consensus coding sequence which is 4752 nt long and encodes a 1583 AA protein (mouse GLI3-FL/mGLI3-FL) (CCDS36603.1)(NP_032156.2). Using Align Sequences Protein BLAST, we aligned amino acid sequences of mouse Gli3 and human GLI3 (Fig. 3). This revealed the biochemical domains of mouse Gli3-FL are 86% conserved in human Gli3-FL. All domains show high similarity with the exception of the transactivation domain (Similarity of human to mouse: GLI3-R domain: 98%, DNA binding domain: 100%, Cleavage site: 95%, CBP binding domain: 79%, TA2 domain: 68%, TA1 domain: 85%, Total transactivation site: 76%). Interestingly all phosphorylation sites from PKA, GSK3β and βTrCP are 100% conserved between mouse and human GLI3-FL (Fig. 2). This is not surprising since multiple studies show similar function of mouse Gli3-R in comparison to human GLI3-R regarding inhibition of HH signaling [113, 117, 118]. Amino acid sequences that are important for biochemical domains are usually conserved between species. Since the human transactivation domain shows relatively low similarity with the mouse transactivation domain of Gli3-FL, this raises the question whether or not this domain is sufficiently defined in humans yet. Hopefully, future studies will further characterize transactivation site in human GLI3 and possibly identify novel interaction partners at this protein locus.

#### Processing of GLI3-FL to GLI3-R

**Fig. 4:**
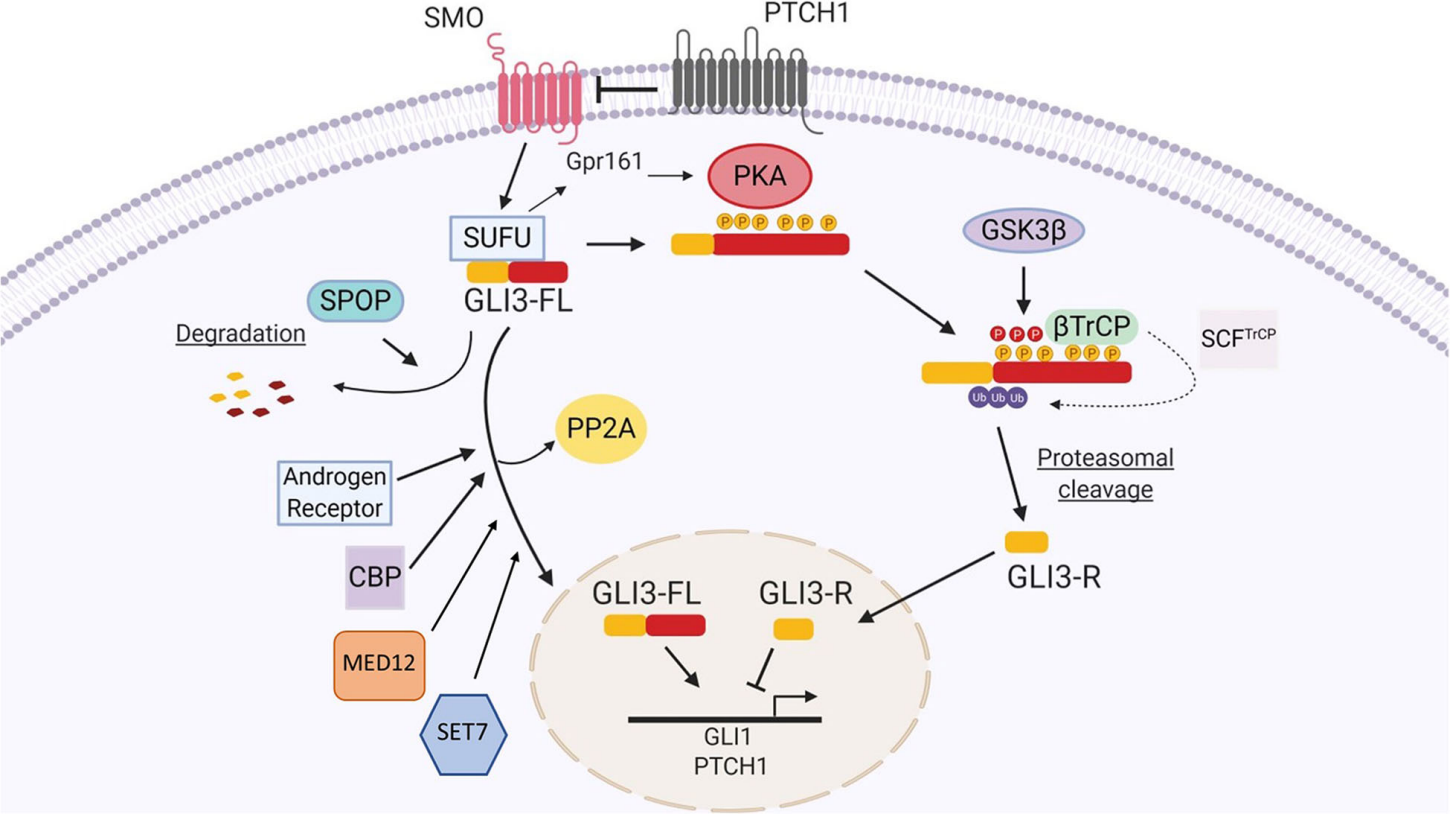
Regulation of GLI3-FL and GLI3-R. GLI3-FL (but not GLI3-R) is proteasomally degraded upon overexpression of SPOP, while binding by CBP, Androgen receptor, MED12, SET7 and release of PP2A stabilizes GLI3-FL and facilitates its translocation to the nucleus. GLI3-R generation is facilitated by SUFU recruitment of Gpr161, which activates PKA. PKA phosphorylates GLI3 at AA residues 849, 865, 877, 907, 980 and 1006. This phosphorylation leads to hyperphosphorylation by GSK3β (AA 841-880) and binding of βTrCP to GLI3. βTrCP then recruits SCF^TrCP^ which ubiquitinates GLI3 leading to proteasomal cleavage and generation of GLI3-R which can translocate to the nucleus to regulate gene expression (Figure created with BioRender.com)

Gli2 and Gli3 show 95% amino acid similarity and both can potentially have HH activator and repressor functions [119]. However, Gli2 proteasomal cleavage is not very efficient and the cleavage product that is produced rapidly degrades while Gli3-R is very stable [120]. Therefore, GLI2 is considered an activator in response to HH signaling while GLI3 is considered a repressor in that context. In the absence of Hedgehog signaling, GLI3-FL is sequestered in the cytoplasm by Sufu (Fig. 2 and Fig. 4) [121]. Sufu recruits Gpr161 which activates Pka and allows it to phosphorylate Gli3 [122]. PKA phosphorylates human GLI3 at serine residues 849, 865, 877, 907, 980 and 1006 (PKA sites: RRXS) (Fig. 2 and Fig. 4) [122]. This leads to additional phosphorylation of GLI3 by GSK3β (at AA 861, 873 and 903)(GSK3β: SXXXpS) [123]. Subsequently, GLI3 binds βTrCP at AA 855, 856, 864 and 894, which recruits Skp1-Cul1-F-box protein-βTrCP complex (SCF^TrCP^) [124] (Fig. 2 and Fig. 4). This results in ubiquitination of GLI3 at lysine residues 773, 778, 784 and 800 by SCF^TrCP^ resulting in GLI3 processing into its repressor form (GLI3-R) (Fig. 2 and Fig. 4) [124]. Binding of βTrCP was also reported to occur at the N-terminus and at the C-terminus, however this binding is PKA-independent (binding sequence: AA 1-395 and 1100-1595) [124]. GLI3-R translocates to the nucleus where it suppresses HH related functions [125].

#### GLI3-FL, stabilization and degradation

It is generally accepted that GLI3-FL is the transcriptional activator form of GLI3 [62, 126, 127]. However, GLI3-FL has been reported to directly interact with the androgen receptor and stimulation of pancreatic cancer cells with the synthetic androgen “R1881” (methyltrienolone) leads to the nuclear translocation of GLI3-FL and subsequent promoter activation of specific GLI consensus sequences [128]. In this study, the androgen receptor was also shown to stabilize GLI3-FL. Despite the role of Sufu in sequestering Gli3 in the cytoplasm to suppress its transcriptional activity, the presence of Sufu has also been shown to stabilize Gli3 protein [129]. In addition, the Speckle-type POZ protein (SPOP) induces proteasomal degradation of GLI3-FL but not of GLI3-R [129], Protein phosphatase 2A (PP2A) directly binds GLI3-FL, and the degradation of PP2A leads to increased nuclear localization of GLI3-FL [127]. GLI3-FL has also been reported to interact with CBP in a phosphorylation-independent manner at AA 827 and 1132 in the C-terminal end of GLI3. This interaction is important for activation of the GLI1 promoter by GLI3-FL and was only detectable in the presence of SHH [62]. In addition, Mediator of RNA polymerase II transcription subunit 12 (MED12) physically interacts with GLI3 at AA 1090-1596 and facilitates gene transcription upon HH stimulation [130]. Finally, SET domain containing 7 (SET7) increases the stability of GLI3-FL and its ability to bind the GLI1 promoter region upon SHH treatment [131]. In summary, multiple studies have investigated the regulation of GLI3-FL in response to canonical HH signaling. Although this information is very valuable, it does not address the possibility that GLI3-FL is regulated in a non-canonical pathway similar to other GLI family members. Therefore, further studies are needed to determine if GLI3-FL regulation is similar in canonical (HH) and non-canonical signaling pathways.

## Role in development, immune system and cancer

### Development

The role of GLI3 in regulating development is frequently described. In addition to its importance in brain and lung development, GLI3 is a key player in the manifestation of GCPS, PHS and Tibial Hemimelia, [8, 132, 133]. These genetic diseases are known for the formation of an extra digit (postaxial polydactyly) consistent with the Gli3^Δ699^ mouse model (mice with mutated Gli3 that have similar phenotypes compared to PHS in humans) [134]. Several mutations of GLI3 which compromise GLI3 function, are tightly linked to these developmental disorders, emphasizing that GLI3 is essential for proper human development. In the following section, we provide a more detailed discussion to further elaborate on the role of GLI3 in development and genetic diseases.

#### GCPS, PHS and tibial hemimelia

Individuals suffering from GCPS develop postaxial polysyndactyly (limb duplication) of the hands, preaxial polysyndactyly of the feet, and macroencephaly (enlarged head). This genetic disorder is caused by a deletion of a region located on chromosome 7p14. Vortkamp et al (1992) identified GLI3 as the protein responsible for this disorder and the related phenotype [135]. That study reports *GLI3* to be present on chromosome 7p13, however, as stated earlier, recent updates correctly identify the location of *GLI3* on chromosome 7p14 [4, 108, 109]. SHH is responsible for limb development in the anteroposterior axis [136]. The cause for the polydactyly phenotype is a mutation within the GLI3 protein which disrupts the equilibrium between GLI3-R and GLI3 activator (GLI3-FL) forms, resulting in the lack of negative regulation of SHH signaling. In GCPS, mutations in GLI3 can occur throughout the whole protein and can vary from missense to splicing mutations, although deletions, insertions and translocations have been documented [137]. N-terminally truncated versions of GLI3 protein, which completely or partially lack the zinc finger DNA binding domain, are common in GCPS. There are also reports where families have a missense mutation in His601Arg which is located near the C-terminal end of the 5 zinc finger DNA binding domain [138]. Another reported mutation in GLI3 protein is located at AA 903-934 which are within the transactivation domain [139]. Thus, GCPS-related mutations in GLI3 occur throughout the whole protein and cannot be reduced to one mutation. In addition, the involvement of other proteins in GCPS cannot be ruled out.

PHS is clinically identified by mesoaxial (central) polydactyly and hypothalamic hamartoma (benign tumor of the hypothalamus) [140]. Mutations related to PHS are usually frameshift or nonsense mutations. These mutations lead to a truncated version of GLI3 which is 691 AA long [8]. Transgenic mice expressing truncated Gli3 (699 AA long) show PHS characteristics and this truncated Gli3 exhibits inhibitory functions similar to Gli3-R in the context of Shh signaling [134]. It can therefore be inferred that mutations in PHS lead to expression of a truncated version of Gli3 similar to Gli3-R, mimicking its function and leading to PHS (probably also due to the lack of Gli3-FL). Kang et al (1997) suggested that PHS was caused by a deletion of a guanosine at nt 2023 in exon 12 of GLI3 [8]. This exon spans the cleavage site for GLI3-FL to be processed into the GLI3-R form. Several additional mutations have been identified and can be found between AA 717-1297 [140]. This spans roughly the beginning and the end of the transactivation domain and includes ubiquitination and phosphorylation sites of GLI3 (Fig. 2).

Another clinical condition involving GLI3 is tibial hemimelia, a genetic disorder which leads to hypoplastic or absent tibia [133]. Deimling et al (2016) reported a deletion of exon 7 of GLI3 upstream of the DNA binding domain and the transactivation domain [141]. This deletion leads to a 30 kDA truncated version of GLI3 that is no longer capable of negative regulation of PTCH1 or GLI1 in response to HH signaling. These studies highlight the important role of GLI3 in limb development.

#### Brain development

In the late 90’s Gli3 was shown to be involved in development of the Dentate gyrus and hippocampus, 2 regions which are responsible for emotional development and memory [142]. In this study Xt^J^ mice show a loss of Homeobox protein Emx1/2 (a transcription factor complex that regulates brain development) expression and show differences in fibroblast growth factor 8 (Fgf8) and bone morphogenetic protein 4 (Bmp4) expression compared to WT mice [142]. Gli3 also plays a role in the development of dorsal telencephalon. In a study that used the extra toes (Xt^J^) mouse model, an impairment in the development of dorsal di-telencephalic junction was reported [143]. This also led to a decrease in the size of the neocortex and missing parts of the hippocampus. Gli3 also controls growth and expansion of the cerebral cortex. A study using Kinesin-like protein Kif3a (Kif3a) mutant mice (where loss of Kif3a leads to the degradation of primary cilia) found a dysregulation of the expansion of cerebral cortex. The loss of primary cilia disrupts Gli3 processing which changes expression of the Shh target genes cyclin D1 and fibroblast growth factor 15 (fgf15) [144]. Petrova et al (2013) reported that the Gli3-R mediates proliferation in the dorsal and ventral subventricular zone [145]. Gli3-R mediates Shh signaling in astrocytes in the forebrain. Gli3 was also shown to be involved in regulating corpus callosum formation by interacting with Slit homolog 2 protein (Slit2) (a protein important for neural development) and therefore regulating Fgf and Wnt/β-catenin expression [146]. In mice, Gli3-R regulates the differentiation of the upper layer of cortical neurons [147]. Furthermore, using Shh^-/-^ Gli3^-/-^ mice embryos, Gli3 was found to play a role in the development of mature oligodendrocytes [148]. Finally, Gli3-R was shown to suppress V0 and V1 interneurons since restoration of motor neurons and V2 interneurons was detected in Shh^-/-^ Gli3^-/-^ embryos in comparison with single Shh^-/-^ knockout embryos [149]. These studies define an important role for Gli3 in brain development and furthers our understanding of the developmental role of this transcription factor.

#### Lung development

There is evidence for a role of Gli3 in the embryonic stages of mouse lung development [150]. High levels of Gli3 expression were reported in E11.5 mouse embryos in the tip of the accessory lobe. In addition, using *Gli3*^*XtJ*^ mice, Gli3 deficiency at E13.5 led to a reduction in size and changes in the shape of three of the five lung lobes [150]. The role of Gli3 in lung development was confirmed by another group who described the impact of Gli2 deficiency in mice lung development to be substantially increased when GLI3 was knocked out as well. They showed that *Gli2*^*-/-*^*Gli3*^*-/-*^ mice embryos lacked lungs, tracheae and esophagi [151]. Therefore, in addition to its role in limb and brain development, Gli3 also regulates lung development.

### Role of GLI3 in the immune system

A role for GLI3 in the immune system has been suggested in several studies [152–156]. Based on current knowledge, the regulatory role of GLI3 in the immune system spans both innate and adaptive immunity. GLI3 has been shown to regulate immune cell development and may play a role in inflammation induced by bacterial infections. A role for GLI3 in the immune system was first described in 2005, in which Gli3 regulates the immune response by modulating T cell development [152]. In this report, Gli3 regulated fetal double negative 1 (DN1) to DN2 transition and the progression of developing thymocytes from double negative (DN) to double positive (DP) state was impaired in the absence of Gli3. This regulatory effect of Gli3 occurs after pre-TCR signaling. These studies suggest that Gli3 is important for T cell development. However, whether this results in a biological effect of Gli3 loss on T cell responses to an immunological challenge remains unknown. The Cluster of Differentiation 155 (CD155; also known as poliovirus receptor) is a member of the immunoglobulin superfamily and binds to the leukocyte adhesion molecule DNAM1. Both of these molecules are expressed on the surface of NK cells [154]. Treatment of cells with the Shh ligand induced CD155 expression [155, 156]. Further analysis indicated the core promoter region of CD155 contains candidate GLI binding consensus sequences which, when mutated, results in reduced promoter activation by both Gli1 and Gli3 [156]. This suggests a role for Gli3 in regulating NK cell activation and function in the immune system via regulation of CD155 expression [156]. A role for Gli3 in fetal B cell commitment was suggested in a study where a dose-dependent reduction in CD19^+^, B220^+^ and CD19^+^ B220^+^ B cell numbers in *Gli3* WT, *Gli3*^*+/-*^ and *Gli3*^*-/-*^ embryos was observed [157]. In addition, the total number of CD93^+^ B cells and the number of ckit^+^ CD127^+^, CD43^+^ CD19^+^ HSA^+^ BP-1^+^ and CD19^+^ HSA^+^ μH^+^ B cell subsets was significantly reduced in *Gli3*^*-/-*^ E18.5 mouse embryos [157]. Taken together, these studies suggest Gli3 as a player in lymphocyte development. However, the biological significance of this reduced lymphocyte populations has yet to be elucidated.

In a study using RAW264.5 mouse macrophages that were challenged with lipopolysaccharide (LPS) to identify novel inducible genes, a muscle specific transcription factor Myoblast determination protein 1 (MyoD) and a major regulator of cardiac development Homeobox protein Nkx-2.5 (Nkx2.5) were reported to be upregulated. More interestingly, the expression of *Gli3*, but not *Gli1* or *Gli2*, was upregulated in response to LPS suggesting a novel mechanism of regulation of *Gli3* by the TLR4 signaling pathway [158]. Although this is the only report to suggest a regulatory role for Gli3 in Tlr4 signaling, it is probable that Gli3 might regulate innate inflammatory effects based on aforementioned studies. Additionally ,in a shRNA library screening during the development of myeloid cells Gli3 was suggested to play a role in cell proliferation [153].

### In cancer

GLI3 regulates various biological processes that are important for cancer cell growth and progression. Several studies found that GLI3 regulates anchorage independent growth, proliferation and migration of cancer cell lines [131, 159, 160]. Studies also reported increased GLI3 expression in patient samples compared with healthy donors [160–162]. A role for GLI3 as a tumor suppressor was reported in medulloblastoma and AML [163, 164]. Although these studies are infrequent, this raises the question of how GLI3 can act as both a tumor initiator and tumor suppressor. The following section summarizes data in which GLI3 was identified as either a pro- or anti-cancerous protein and discusses what is known and what needs to be further investigated to delineate the role of GLI3 in cancer.

In glioblastoma, GLI3 was downregulated upon anti-cancer drug treatment [165]. In this study the focal adhesion kinase (FAK) autophosphorylation inhibitor ‘Y15’ effectively decreased tumor growth of the glioblastoma cell lines DBTRG and U87, especially in combination with temozolomide (a chemotherapeutic agent that induces G2/M arrest and apoptosis). In a microarray gene profiling analysis where glioblastoma cell lines DBTRG and U87 were treated with ‘Y15’ in combination with temozolomide, the expression of GLI3 was downregulated [166]. This downregulation was not visible in the presence of either drug alone suggesting that downregulation of GLI3 is a result of synergistic inhibitory effect of this drug combination [166]. It can be inferred that GLI3 downregulation is involved in the synergistic anti-tumor effect of both treatments, however the molecular mechanism resulting in this downregulation remains unclear.

In the earlier described PHS, mutations in GLI3 cause formation of polydactyly of the limbs [167]. However patients suffering from PHS also have a high recurrence of hypothalamic hamartoma, suggesting a role of GLI3 in regulating benign brain tumor formation [167–169]. In a study by Saitsu et al (2008), two somatic mutations in GLI3 in hypothalamic hamartoma patients were identified and showed reduced GLI promoter activity from the floor plate enhancer HNF3b in C3H10T1/2 cells [170]. This suppressor activity of mutated GLI3 might be related to hypothalamic hamartoma formation, however further studies are necessary to confirm the role of GLI3-R and HH signaling in this tumor development.

In addition to the involvement of GLI3 in hamartoma, GLI3 plays a role in Oral squamous cell carcinoma (OSCC) [159]. PCR array results comparing CD44^high^ vs CD44^low^ OSCC cell populations using SCC4 and SCC9 cells revealed a greater than 6-fold increase in GLI3 expression in the CD44^high^ cancer stem cell subtype [159]. This upregulation of GLI3 expression was also confirmed *in vivo* in OSCC patient samples [159, 171]. In addition, a significant reduction in proliferation and invasion was observed *in vitro* and a positive correlation between GLI3 expression and tumor size was confirmed *in vivo* [159]. In this publication GLI3 knockdown had no effect on apoptotic cell death and there was no link between GLI3 expression and metastasis or blood lymphatic or perineural invasion. Although patients with high GLI3 expression seem to survive less, data presented in the Kaplan–Meier blot was not statistically significant. These reports show that GLI3 regulates cancer stem cells in OSCC by regulating relevant markers for EMT. Additionally, GLI3 regulates cell survival and invasion and correlates with tumor size *in vivo*. However, whether GLI3-FL or GLI3-R are regulating this effect, is not clear and the involvement of classical HH signaling has not been investigated. Since HH signaling is involved in regulating cancer stem cell subtypes in several cancers, it is plausible that SHH regulates this subtype partially via GLI3 in a paracrine manner [15]. However, additional studies are needed in order to reach this conclusion. Since cancer stem cells are resistant to most chemotherapy due to irregular cell division, GLI3 might be a potential target to sensitize those cells to chemotherapeutic agents. For example, since GLI3 negatively regulates S100A9 in OSCC cancer stem cells and this has been shown to induce cell death in cancer cell lines [172], targeted depletion of GLI3 in OSCC might be an effective strategy in the treatment of OSCC.

In addition to OSCC cancer stem cells, gastric cancer stem cells, that were sorted based on CD44^+^ CD24^+^ surface expression, showed 80-fold higher GLI3 mRNA expression than the CD44^lo^ CD24^lo^ phenotype which positively correlated with mRNA expression of SHH and PTCH1 [161]. Since multiple studies report the involvement of SHH in gastric cancer [173, 174], it is possible that GLI3 is mediating SHH-induced effects. However additional studies are needed to confirm the specific regulatory role of GLI3 in gastric cancer development and progression. Additional evidence supporting a role for GLI3 as an oncogene was provided by Li et al (2018) who used bladder cancer tissue samples to show higher GLI3 expression in tumor samples vs benign tissue by performing microarray gene expression analysis [160]. Interestingly, GLI3 was identified as a prognostic factor for poor survival in patients suffering from bladder cancer and, consistent with aforementioned studies, GLI3 was shown to regulate cell proliferation, invasion, migration and proteins involved in EMT such as E-cadherin and N-cadherin [131, 159, 160]. miR-7-5p was identified as a negative regulator of GLI3 in bladder cancer [160]. However, no experiments were performed to identify possible downstream targets of GLI3. Additionally, given the role of GLI3 in regulating cancer stem cells in OSCC and gastric cancer, it might be useful to determine the role of GLI3 in regulating bladder cancer stem cell characteristics and survival as well.

Post translational modifications of GLI3-FL (but not GLI3-R) by SET7 has been reported to increase the stability of GLI3-FL and its ability to bind the promoter region of GLI1 upon SHH treatment [131]. SET7 lysine 436 (K436) and -K595 dependent methylation of GLI3-FL regulates cell viability and colony formation of the non-small cell lung cancer (NSCLC) cell line A549 as well. Additionally, A549 xenografts in mice, expressing GLI3-FL with mutations in SET7 methylation sites, showed less migration, invasiveness and decreased tumor volume in comparison to mice injected with A549 xenografts expressing WT GLI3. This supports the oncogenic role of GLI3 and provides information on post-translational modification-dependent tumorigenicity of GLI3 in NSCLC. Furthermore, targeting SET7 may be a viable therapeutic strategy to decrease GLI3’s oncogenic effect in NSCLC as a strategy to antagonize SHH-dependent signaling.

In colon cancer, GLI3 transcripts were significantly higher than in healthy tissue [175]. Treatment of colon carcinoma cell lines RKO and LOVO with GLI3 siRNA led to a significant decrease in cell proliferation. Furthermore, GLI3 levels inversely correlated with p53 levels within those cell lines. Additional evidence from *in vitro* experiments showed that siGLI3 treatment decreased p53 and MDM2 interaction and reduced ubiquitination of p53 [175]. However, it is unclear if this effect is due to GLI3-FL or GLI3-R and warrants further investigation [175]. In another study, GLI3-FL (but not GLI3-R) induced more anchor-independent growth in the human colorectal cancer cell lines HCT116, HT29, SW480 and DLD-1 which was also visible upon SHH stimulation suggesting GLI3 regulation of colony formation occurs in a paracrine manner as well [12]. In this study, the oncogenic role of GLI3 in solid tumors was further validated when GLI3-FL-overexpressing DLD-1 and HT29 mouse xenografts showed significantly increased tumor formation compared to control cells. Interestingly, GLI3 did not seem to be affected by any known cancer-related signaling molecules such as p53, WNT or MAPK signaling [12].

In pancreatic cancer cells such as PANC-1, targeting GLI3 with siRNA reduced cell viability. Additionally, siRNA mediated GLI3 knockdown appeared to sensitize PANC-1 cells to cyclopamine treatment *in vitro* [176]. Since cyclopmine’s reduction of cell viability is due to the initiation of pro-apoptotic cell machinery, the question arises as to what degree GLI3 is involved in regulating the apoptotic pathway in pancreatic cancer cells.

In a more recent study, GLI3-FL was suggested to be involved in the induction of castration-resistant prostate cancer (GRPC) through a mutated form of MED12 which is common in prostate cancer [177]. MED12 negatively regulates GLI3-FL activation role by directly interacting with GLI3 at AA 1090-1596 [130]. Due to a mutation in MED12, its constraining activity toward GLI3 is inactive, which leads to activation of SHH-GLI3 related gene expression in the absence of androgen [177]. Since a common therapy for prostate cancer is androgen depletion, and in the presence of mutated MED12 SHH induced GLI3 gene expression is activated when androgen is absent, new or additional therapeutic approaches might be necessary to reduce the risk of prostate cancer relapse. Furthermore, the question remains whether there is a direct interaction between GLI3 and MED12 in prostate cancer. Although this interaction between MED12 and GLI3 is probable, there are no reports of MED12 interacting with GLI3 to regulate HH related gene expression. In addition, it also remains unclear if MED12 is required for GLI3 phosphorylation. Further research is needed to elucidate the mechanism of MED12-GLI3 regulation.

It can cautiously be suggested that GLI3 may play an oncogenic role in germ cell tumors. In a study by Kuleszo et al (2017), eight germ cell tumors from children were used to perform genomic profiling and GLI3 was identified to have additional copies of its gene in the germ cell tumor group compared with healthy individuals [178]. Although no experiments were performed that directly identify GLI3 as an oncogenic protein in germ cell tumors, the higher GLI3 copy numbers in germ cell tumors is indicative of more HH activation and SHH related gene expression which is known to drive tumor growth and progression. The presence of higher copies of SMO and SHH, in addition to lower copy numbers of PTCH1 (which inhibits SMO activity) in germ cell tumors are another supporting fact of the involvement of HH signaling in germ cell cancer formation.

In addition to regulating proliferation and invasiveness in cancer, GLI3 was also reported to modulate vacuole membrane protein (VMP1), a known regulator of autophagy [179]. Lo Ré et al (2012) reported that oncogenic KRAS (KRAS^G12D^) leads to activation of VMP1 by signaling through PI3K-AKT1-GLI3. In this pathway, GLI3 physically interacts with p300 which facilitates binding of GLI3 to the promoter region of VMP1. *GLI3* mRNA expression was upregulated upon transfection with oncogenic KRAS^G12D^ and co-expression of GLI3 with constitutively active forms of PI3K and AKT1 showed higher VMP1 promoter activity. These studies show the regulation and requirement for GLI3 in autophagy; however, it is unclear which transcription factor is involved in regulating GLI3 expression downstream of AKT1. In addition, it would be interesting to determine if post translational modifications of GLI3 are required for binding and activating VMP1 promoter activity. This could be accomplished by targeting known regulators of GLI3 such as PKA or GSK3β, in addition to activating this pathway. Additionally, it is unclear if GLI3-FL or GLI3-R is responsible for the regulation of VMP1 and the biochemical domains mediating the interaction between GLI3 and p300. Depending on the binding site for the interaction between GLI3 and p300, this may determine if GLI3-FL or GLI3-R regulates the KRASG12D-PI3K-AKT1-VMP1 pathway.

In addition to the oncogenic role of GLI3 in solid tumors, GLI3 was shown to play a similar role in hematologic malignancies. Elevated *GLI3* expression was reported in Hodgkin lymphoma cell lines [180]. This was validated using immunohistochemistry in primary patient biopsies where GLI3 was found in Hodgkin/Reed Sternberg cells. GLI3 was also suggested to pay a role in Diffuse large B cell lymphoma (DLBCL). This is an aggressive lymphoma that is divided into 2 subtypes based on gene expression profiling studies: activated B-cell (ABC) and germinal center B-cell (GCB) subtypes. *GLI3* expression was examined in a cohort of DLBCL cell lines and was found to be elevated in cell lines belonging to the GCB DLBCL subgroup [181]. Using gene expression datasets using DLBCL patient samples, the authors also report increased *GLI3* expression in the GCB subtype. Knockdown of GLI3 reduced cell proliferation suggesting that GLI3 promotes cell growth in GCB DLBCL. Further characterization of the biological role of GLI3 in these cancers will enhance our understanding of the biological significance of GLI3.

Although rare, anti-cancerous activity of GLI3 has been reported as well. GLI3 was found to be present in 94% of neuronal differentiation and glial and neuronal differentiation medulloblastoma, while it could not be detected in any of the differentiation-free medulloblastoma [163]. Additionally, patients with differentiation free medulloblastoma showed significantly less survival. This suggests a role for GLI3 as a prognostically favorable factor in medulloblastoma. The role of SHH in medulloblastoma has been reported and therapeutic success has been shown after inhibiting HH signaling [182, 183]. In theory, as a result of inhibition of HH signaling, GLI3-R should accumulate within the cell and negatively regulate HH-related gene expression and therefore cancer growth. However, whether the presence of GLI3-R form specifically correlates with a higher overall and event free survival has yet to be determined. Additionally a number of studies have reported that GLI transcription factors can be regulated in a HH independent manner [80, 85, 95, 105, 184–187]. Therefore, this raises the possibility that GLI3 may regulate gene expression in a HH-independent manner to mediate its anti-cancerous functions.

Another study reported GLI3 as a tumor suppressor protein using bone marrow from AML patients. Both GLI3-FL and GLI3-R were shown to be expressed at significantly lower levels in AML patient samples [164]. Based on their data, the absence of GLI3 in AML is due to hypermethylation of its promoter region. Global demethylation showed an increase in protein expression of both GLI3-FL and GLI3-R *in vitro* and *ex vivo* which correlated with decreased proliferation of the AML cell lines (K562 and KG1a) and primary AML blasts and with mouse survival after K562 xenograft transplantation. This increase in GLI3-FL and GLI3-R was also visible when AML cell lines were treated with the HH inhibitor PF-04449913 (a SMO inhibitor). Therefore, it might be worth investigating whether PF-04449913 might regulate demethylation of GLI3 promoter in its reported synergistic effect with decitabine in AML [164]. A cross-talk between GLI3-R, AKT1 and ERK1/2 protein has also been reported. Overexpression of GLI3-R negatively correlated with AKT1 but positively correlated with ERK1/2 [188]. AKT1 was reported to be important for GLI3-R effect on negatively influencing cell proliferation [188]. However, it was not determined if GLI3-R regulates proliferation through ERK1/2 as well. ERK1/2 is known to activate MAPK pathway however to what degree GLI-R is involved in this pathway has not been investigated. It might also be of interest to determine the role of GLI3-AKT1 axis in response to HH inhibitor treatment such as PF-04449913 or cyclopamine and if demethylation increased the activity of this pathway.

Most studies that have investigated GLI3 in cancer described the cancer supporting role of GLI3 to occur by positively regulating proliferation, survival and invasiveness. Reports suggesting GLI3’s tumor suppressive role have been rare but raise the question where the contradictory attributes of GLI3 in cancer come from. It is possible the role of GLI3 in cancer is tissue specific. In addition, the majority of studies discussed in this part of the review, describe a role for total GLI3 levels in regulating cancer cell growth and progression. However, it is unknown if it is GLI3-FL or GLI3-R that regulates these effects. This is possibly hindered by the lack of antibodies that reliably identify GLI3-FL or GLI3-R (Matissek et al, unpublished observation). Studies that focused on these two forms of GLI3 have suggested that GLI3-FL induces cancerous behavior while GLI3-R reduces cancer associated attributes [12, 131, 164, 177]. However, to date, there is only one study that solely compares the effect of GLI3-FL vs GLI3-R on cancerous characteristics [189]. In this study, GSK3 beta activity was increased which induced higher concentrations of GLI3-R and an anti-cancerous effect.

Based on previous mentioned work by Chaudhry et al (2015), hypermethylation of GLI3 promoter region leads to a loss of GLI3 and tumor suppressor function in AML [164]. In addition to epigenetic modifications that suppress GLI3 tumor function, it might also be that a disruption of GLI3-FL/GLI3-R equilibrium within the cell leads to a cancerous phenotype and tumor growth. Different expression and activation levels of GLI3-FL regulators (SPOP, androgen receptor, PP2A, CBP, SET7, MED12) or regulators of GLI3-R (PKA, GSK3β, βTrCP) in cancer cells in comparison to healthy cells might also promote cancer cell growth and survival.

GLI3 levels also positively correlated with higher expression of cancer stem cell markers and shRNA-mediated knockdown of GLI3 led to a decrease in the CD44^high^ population (cancer stem cells) while the CD44^low^ population was unaffected in OSCC [159]. The small cancer stem cell population within a tumor has been shown to be very potent in inducing tumor growth in OSCC [190]. Therefore, GLI3’s tumorigenic role may be due to its regulation of cancer stem cell survival. However, whether GLI3-FL or GLI3-R regulates this effect is also not fully explored. There are multiple HH inhibitors (cyclopamine, PF-04449913, GDC0449, Saridegib, Vismodegib) that showed promising results in fighting HH-driven cancers. These inhibitors target SMO to inhibit HH-related gene expression. However, since HH-independent regulation of GLI transcription factors has been reported, this might rescue the effects induced by SMO inhibition [191]. Therefore, therapies such as GANT61, which inhibit binding of GLI1 and GLI2 transcription factors to DNA, are promising since they act directly on GLI but not on SMO [192]. GLI3 expression was not affected by GANT61, therefore, targeting modulators of GLI3-R by making them more active, might decrease PTCH1-SMO-independent HH gene expression and increase the therapeutic effect of GANT61 treatment [98]. Furthermore, a thorough understanding of the signaling pathways that regulate GLI3 independent of HH will allow therapeutic targeting of GLI3 by targeting other molecular regulators of this protein.

### MicroRNA (miRNA) and GLI3

As described earlier, GLI3 can interact with several different proteins, an interaction that is required for either stabilization or degradation of GLI3 protein. However, proteins are not the only molecules involved in regulating GLI3. Several lines of evidence have been reported in which *GLI3* is regulated at the RNA level by microRNAs which are described below. MicroRNAs were reported to bind complementary sequences of GLI3 RNA and negatively regulate its gene expression.

#### In cancer, liver fibrosis and spermatogenesis

GLI3 was shown to be regulated by miR-7-5p in bladder cancer tissue. A significant upregulation of GLI3 in bladder cancer was reported by microarray gene expression profiling. Using the online software (TargetScan, PITA, miRanda) the authors found that miR7-5p was the most potent candidate for GLI3 regulation and later confirmed its regulatory role using *in vitro* experiments. In those studies, a physical interaction between miR-7-5p and GLI3 was identified (GLI3 interaction site: GUCUUCCA), which leads to a negative regulation of GLI3 and to a less cancerous phenotype of bladder cancer cells [160].

In other studies, miR494 and 506 were shown to regulate tGLI3 oncogenic function in pancreatic and cervical cancers [162]. In pancreatic cancer, GLI3 was regulated by miR-494 [162]. *GLI3* mRNA expression was significantly upregulated in tissue from bladder cancer patients and there was an inverse correlation between GLI3 and miR-494. Furthermore, miR-494 negative regulation of GLI3 was confirmed *in vitro* in which inhibition of GLI3 by miR-494 led to a decrease in cell viability and cancer cell migration [162]. A similar negative regulation of GLI3 was also detected by miR-506 in cervical cancer [193]. This was reported in both primary patient biopsy samples and in cervical cancer cell lines using western blotting. GLI3 protein was increased upon miR-506 inhibition which led to reduced apoptosis and increased proliferation. In addition, mice xenografts with Casci cell stably expressing sh-miR-506 showed significantly higher tumor weights in comparison to control mice.

In addition to their role in tumorigenesis, micro RNAs appear to regulate GLI3 in liver fibrosis. Both miR-152 and miR-378a-3p have been shown to negatively regulate GLI3 in liver fibrosis *in vitro* and *in vivo* [194, 195]. These studies used the LX-2 hepatic stellate cells where they overexpressed miR-152 or miR-378a-3p and found a reduction in GLI3 mRNA and protein expression. Using rodent models of liver fibrosis where fibrosis was chemically induced with CCI4 in mice, *GLI3* expression was increased (miR378a-3p downregulated) in tissue isolated from animals suffering from liver fibrosis suggesting GLI3 positively regulates liver fibrosis.

The role of GLI3 in spermatogenesis in mice was established in 1997 [196]. However, in 2016, a role for miR-133b in regulating Sertoli cells by targeting GLI3 was discovered [197]. miR-133b was significantly upregulated in Sertoli cells from patients suffering from Sertoli-cell-only syndrome (SCOS) and using TargetScan, GLI3 was identified as a potential target for miR-133b. This was confirmed by *in vitro* experiments showing the regulatory role of miR-133b in which an increase in miR-133b led to reduction of GLI3 mRNA and protein expression [197].

## Conclusions

The importance of HH signaling in many biological pathways such as those related to development, cancer and inflammation, has been frequently reported. The involvement of HH signaling in positive regulation of these biological functions has focused on GLI1 and GLI2 which are known activators of HH signaling. Their role as a positive regulator in non-canonical HH pathways has been frequently described as well. However, based on this review, GLI3 also shows high potential in regulating non-canonical pathways positively. In addition to early reports of the role of GLI3 in the development of the brain, lungs, sperm and in genetic diseases, the biological significance of GLI3 was also suggested in diseases such as liver fibrosis, cancer, and in the immune system. In cancer, GLI3’s behavior seems to be bipolar since it was linked to cancer promoting and inhibitory effect in cells which can be explained by GLI3-FL activator and GLI3-R inhibitory function in gene expression. In addition, recent research shows that negative regulation of GLI3 through microRNA reverses cancerous behavior which emphasizes GLI3 as a potential oncogenic target in cancer therapy. Future studies focusing on determining the role of GLI3 in the immune response and in cancer will clarify its biological significance and lay the foundation to target this molecule to reprogram immune and cancer cells.

## Data Availability

Not applicable

## Abbreviations

ABC: Activated B cell
AML: Acute Myeloid Leukemia
Bmp4: Bone morphogenetic protein 4
Boc: Brother of Cdo
CBP: CREB-binding-protein
CD155: Cluster of differentiation 155
Cdo: CAM-related/downregulated by oncogenes
CDS: Consensus coding sequence
Ci: Cubitus interruptus
Ck-1/CK-1: Casein kinase 1 (mouse/human)
CK2a: Casein kinase 2a
CLL: Chronic lymphocytic leukemia
Cos-2: Costal-2
Dhh/DHH: Desert Hedgehog (mouse/human)
DLBCL: Diffuse large B cell lymphoma
DN: Double negative
DNAM1: CD226, Cluster of differentiation 226
Dnchc2: Dynein cytoplasmic heavy chain 2
DP: Double positive
DYRK1/DYRK2: Dual specificity tyrosine-phosphorylation-regulated kinase ½
EMT: Epithelial-to-mesenchymal transition
Emx1/2: Homeobox protein Emx1/2
Evc/Evc2: Ellis-van Creveld syndrome protein/Ellis-van Creveld syndrome protein 2 complex
FAK: Focal adhesion kinase
Fgf15: Fibroblast growth factor 15
Fgf8: Fibroblast growth factor 8
Fu: Fused
Gas1: Growth-arrest-specific 1
GCB: Germinal center B cell
GCPS: Greig cephalopolysyndactyly
Gli3-FL/GLI3-FL: Full length GLI3 protein (mouse/human)
Gli3-R/GLI3-R: Repressor form of GLI3 protein (mouse/human)
Gpr161/GPR161: G-protein coupled receptor 161 (mouse/human)
Gsk3β/GSK3β: Glycogen synthase kinase 3 beta (mouse/human)
Hh/HH: Hedgehog (mouse/human)
Hhip: Hh interaction protein
HSC: Hedgehog Signaling Complex
IFT: Intraflagellar transport
Ift172: Intraflagellar transport protein 172
Ihh/IHH: Indian Hedgehog (mouse/human)
Kif3a: Kinesin-like protein Kif3a
KIF3A: Kinesin-like-protein KIF3A
Kif7: Kinesin-like protein Kif7
MyoD: Myoblast determination protein
Nkx2.5: Homeobox protein Nkx-2
OSCC: Oral squamous cell carcinoma
PHS: Pallister-Hall Syndrome
Pka/PKA: Protein kinase A (mouse/human)
PKC: Protein kinase C
PP2A: Protein phosphatase 2A
Ptc: Patched (*Drosophila*)
Ptch1/PTCH1: Patched 1 (mouse/human)
Rab23: Ras-related protein Rab23
SCFβ^TrCP^: Skp1-Cul1-F-box protein – βTrCP complex
SCOS: Sertoli-cell-only syndrome
SET7: SET domain containing 7
Shh/SHH: Sonic Hedgehog (mouse/human)
Slit2: Slit homolog 2 protein
Smo/SMO: Smoothened (mouse/human)
SPOP: Speckle-type POZ protein
Sufu/SUFU: Suppressor of Fused (mouse/human)
TA1: Transactivation domain 1
TA2: Transactivation domain 2
TME: Tumor microenvironment
VMP1: Vacuole membrane protein 1
βTrCP: Beta-Transducing Repeat containing Protein
